# ^68^Ga-DOTATOC Uptake by Stellate Ganglia, Mimicking a Right Cervical Metastasis of Neuroendocrine Tumors: A Case Report

**DOI:** 10.3390/jcm13237413

**Published:** 2024-12-05

**Authors:** Jules Tianyu Zhang-Yin, Emmanouil Panagiotidis

**Affiliations:** 1 Department of Nuclear Medicine, Clinique Sud Luxembourg, 6700 Arlon, Belgium; 2 Department of Nuclear Medicine, Centre National PET, 4 Rue Ernest Barblé, L-1210 Luxembourg, Luxembourg; 3 Nuclear Medicine, Theageneio Cancer Center, 546 39 Thessaloniki, Greece; m_panagiotidis@yahoo.com

**Keywords:** stellate ganglia, ^68^Ga-DOTATOC PET/CT, pitfall, neuroendocrine tumors

## Abstract

**Background:** ^68^Ga-DOTATOC PET/CT is a functional imaging modality that has revolutionized the evaluation of well-differentiated neuroendocrine tumors (NETs) by targeting somatostatin receptors. This technique has largely replaced conventional gamma camera imaging with 111In-labeled octreotide due to its superior sensitivity and resolution. While the physiologic distribution, normal variations, and common pitfalls associated with ^68^Ga-DOTATOC imaging are well documented, rare but clinically significant pitfalls can still occur. **Methods:** We present a case highlighting one such pitfall: focal ^68^Ga-DOTATOC uptake at the cervicothoracic junction, specifically within the stellate ganglia, which mimicked metastatic involvement of a NET. **Results:** Initially, the uptake was interpreted as a potential right cervical metastasis. To clarify this finding, a follow-up ^68^Ga-DOTATOC PET/CT was performed, which demonstrated no evidence of cervical metastases, thereby confirming the initial uptake as a physiologic variation rather than pathological activity. This case underscores the dynamic variability of ^68^Ga-DOTATOC uptake within the stellate ganglia in the same patient over time. On occasion, the intensity of physiologic uptake in these structures can be pronounced enough to mimic metastatic disease, posing a diagnostic challenge. **Conclusions:** Awareness of this rare phenomenon is essential to avoid misdiagnosis and unnecessary interventions.

## 1. Introduction

Neuroendocrine tumors (NETs) are an uncommon and diverse group of malignancies that most frequently originate in the gastro-entero-pancreatic system and the lungs. A hallmark of these tumors is the overexpression of somatostatin receptors (SSTRs) on their cell membranes, particularly the type 2 receptor subtype [1]. This biological characteristic serves as the basis for SSTR-targeted imaging and therapy.

^68^Ga-DOTATOC positron emission tomography–computed tomography (PET/CT) has become a cornerstone in the diagnosis and management of NETs, offering significant advantages over traditional imaging modalities such as 111In-octreotide scintigraphy [2]. Compared to conventional methods, ^68^Ga-DOTATOC PET/CT provides superior sensitivity, resolution, and accuracy, facilitating better localization of primary and metastatic disease, as well as improved treatment planning [2].

Despite its established efficacy, the interpretation of ^68^Ga-DOTATOC PET/CT images is not without challenges. Misinterpretation can arise from physiological uptake, normal variations, or rare pitfalls, which can mimic pathological findings and lead to diagnostic errors. A mastery of these potential pitfalls is critical for ensuring accurate assessment and optimal patient management, emphasizing the need for thorough familiarity with the nuances of this advanced imaging modality.

## 2. Case Report

A 47-year-old female patient was referred to our institution for a ^68^Ga-DOTATOC PET/CT scan to evaluate her neuroendocrine tumor with diffuse liver metastases.

The patient presented to her primary care physician with complaints of non-specific abdominal discomfort and intermittent diarrhea over the past three months. Furthermore, the patient reported an unexplained weight loss of approximately 3 kg over the past three months, despite no significant change in appetite. There were no indications of jaundice or overt hypoglycemic episodes. Her past medical history was unremarkable, and she did not take any regular medications, except a daily proton pump inhibitor (omeprazole) for occasional acid reflux.

Her symptoms were initially attributed by her general practitioner to irritable bowel syndrome and stress from work, but with the persistence of symptoms, further evaluation was performed in the hospital.

Blood tests showed moderate elevations of liver enzymes with serum glutamic oxaloacetic transaminase at 130 U/L (normal: 7–56 U/L), serum glutamate pyruvate transaminase at 104 U/L (normal: 10–40 U/L), and alkaline phosphatase at 180 U/L (normal: 44–147 U/L). The serum calcium level was 9.9 mg/dL (normal: 8.5–10.5 mg/dL), and the parathyroid hormone (PTH) was 46 pg/mL (normal: 10–65 pg/mL).

There were also significant elevations in tumor markers, with chromogranin A at 3000 ng/mL (normal: <100 ng/mL). In regard to other biochemical markers, the results indicated a gastrin level of 79 pg/mL, which is within the reference range of 0–100 pg/mL. Similarly, glucagon levels were 147 pg/mL, falling within the normal range of 50–200 pg/mL. The somatostatin level was observed to be 80 pg/mL, which is within the reference range of 10–100 pg/mL. Furthermore, the 5-hydroxyindoleacetic acid (5-HIAA) concentration in a 24 h urine collection was reported at 5 mg/24 h, which is within the normal range (reference: 2–8 mg/24 h).

The abdominal ultrasound and contrast-enhanced CT scan each showed multiple hyperechoic lesions in the liver, suspicious for metastases, and a 2.5 cm mass in the head of the pancreas, suspicious for a primary tumor. There was no lymph node involvement on the CT scan.

The biopsy results of a 4 cm liver metastasis showed a G1 pancreatic NET with a Ki67 of 2%.

Treatment with somatostatin analogs was carried out just after the ^68^Ga-DOTATOC PET/CT.

^68^Ga-DOTATOC was administered intravenously at a dose of 2 MBq/kg of body weight, followed by a 60 min uptake period. Fasting before the administration was not required. The ^68^Ga-DOTATOC PET/CT scan covered a field of view from the skull to the mid-thigh and was performed using a Gemini VEREOS PET/CT system (Philips Medical System, 595 Miner Rd. Cleveland, OH, USA). The imaging process began with a CT acquisition (120 kVp, 80 mA.s) and was followed by the PET acquisition, which was conducted in 3D mode with time-of-flight determination at 2 min per bed position.

**Figure 1:**
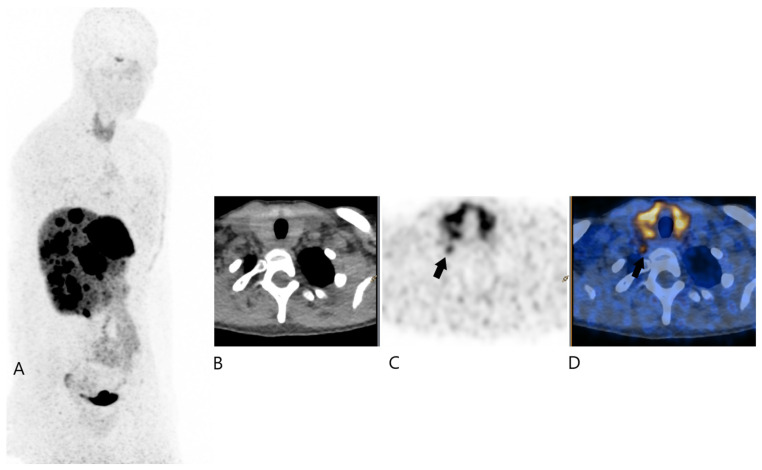
^68^Ga-DOTATOC PET/CT 1 (Image (**A**) for MIP, (**B**) for transaxial PET, (**C**) for transaxial CT, and (**D**) for transaxial fused image); black plain arrows showing the moderate uptake of the right cervical focus.

On this exam, it was shown that highly intense uptake was seen in diffuse bilobular liver metastases. There was also a notable uptake (SUVmax 4.9) observed in the cervicothoracic region of the right lower neck, specifically at the level of the cervicothoracic junction. This uptake was located anterior to the transverse process of the C7 vertebra, inferior to subclavian artery, superior to the neck of the first rib and just behind the right thyroid lobe (Figure 1).

Its uptake is clearly inferior to that of liver metastases and its morphological character is not very lymph node-like. In fact, it appeared as a small and slightly elongated soft tissue structure suggestive of a stellate ganglion and as part of the sympathetic nervous system.

**Figure 2:**
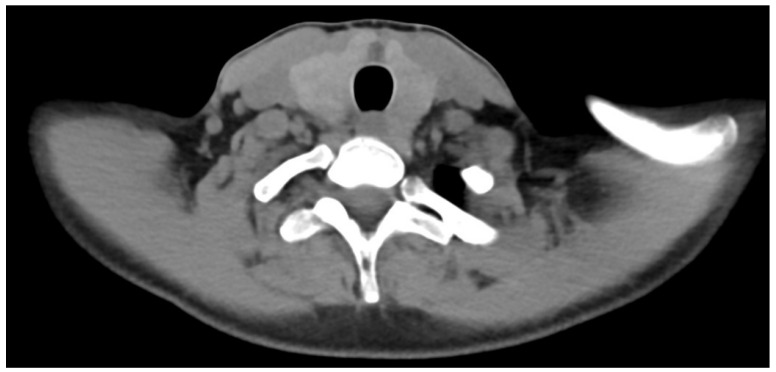
Diagnostic transaxial CT.

**Figure 3:**
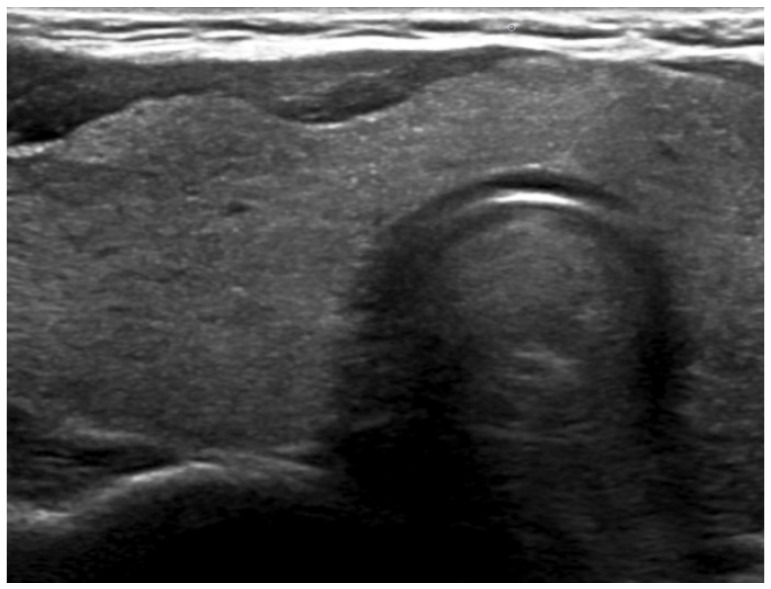
Ultrasound in the region of the right lobe of the thyroid gland.

In order to exclude any hypothesis of cervical metastasis, a diagnostic CT scan with better spatial resolution and a cervical ultrasound were performed (Figure 2 and Figure 3), and no lymph node involvement was found. Lymph node involvement was therefore definitively ruled out in this patient.

**Figure 4:**
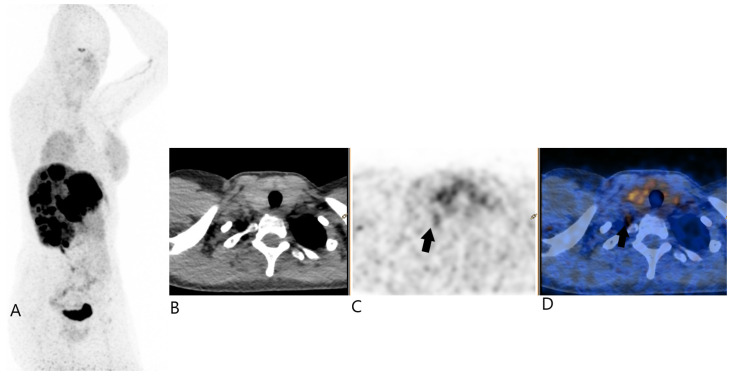
^68^Ga-DOTATOC PET/CT 2 (Image (**A**) for MIP, (**B**) for transaxial PET, (**C**) for transaxial CT, and (**D**) for transaxial fused image); black plain arrows showing the very mild uptake of the right cervical focus.

The patient continued treatment with non-radioactive somatostatin analogs. Three months later, a repeat ^68^Ga-DOTATOC PET/CT scan was performed, 27 days after the previous treatment by somatostatin analog therapy, showing stable disease for liver metastasis and the regression of uptake in this right cervical focus (SUVmax 3.2). The shape was also more elongated and thus more classic to a stellate ganglion (Figure 4).

## 3. Discussion

Pancreatic neuroendocrine tumors (PNETs) are rare, with an annual incidence of less than 1 in 100,000 individuals. Despite their rarity, they comprise 20–30% of digestive NETs in Western countries, ranking among the most common NETs after those in the small intestine or rectum [3,4]. Their true incidence is likely underestimated due to their indolent and often asymptomatic nature, frequently discovered incidentally during imaging or surgery for unrelated conditions. Retrospective epidemiological studies reveal a 5-fold increase in PNET incidence over the past 20 years, now at 0.6 cases per 100,000 population annually [3]. This rise is attributed to advances in diagnostic imaging, histopathology, and molecular biology, enabling better detection and classification. Improved survival rates due to earlier diagnosis and effective treatments have also contributed to their increased prevalence [4,5]. However, the incidence of metastatic PNETs has remained stable, highlighting that the growth primarily reflects the improved detection of primary, incidental PNETs.

PET imaging with ^68^Ga-labeled somatostatin analogs is increasingly replacing ^111^In-pentetreotide scintigraphy as the primary imaging method for diagnosing, staging, and restaging pancreatic NETs. This shift is due to the higher sensitivity, better resolution, and more accurate localization offered by ^68^Ga-labeled PET imaging [6,7]. This recent imaging modality is a powerful tool in the management of patients with well-differentiated NET [7], and more especially the ^68^Ga-DOTATOC PET imaging has been used clinically in Europe [8]. The diagnostic target of ^68^Ga-DOTATOC is not a metabolic pathway, but the pathological overexpression of SSTRs (mainly type 2 and 5). One of its various advantages over scintigraphy is the possibility of performing semi-quantitative analysis. It has also been shown to have prognostic value through functional tumor burden [9] and may be useful in pancreatic NETs with paraneoplastic Cushing’s syndrome [10]. A knowledge of normal physiologic biodistribution and potential pitfalls in interpretation is essential in the accurate interpretation of these studies.

The stellate ganglia, also known as the cervicothoracic ganglion, is formed by the inferior cervical and first thoracic ganglia and is located just anterior to the head of the first rib. It receives input from the paravertebral sympathetic chain and provides sympathetic efferent to the upper limbs, head, neck, and heart [11]. It is present in 80% of people [12]. The ganglion’s size can vary but usually measures around 1–2 cm in length. It may be challenging to identify on a standard CT scan due to its small size and similarity in density to surrounding soft tissues. However, in some cases, with the use of contrast-enhanced CT or when there is increased uptake in PET imaging, its location and morphology can be better delineated [13].

The stellate ganglia expressed SSTRs and their function was to act directly on the high-voltage-dependent Ca^2+^ channel via the Galpha2 protein to block Ca^2+^ currents [14,15]. The presence of SSTR in the stellate ganglion has been documented in several animal species, including the pig and the rat, as well as in humans [16,17,18].

To the best of our knowledge, this is the first case report of ^68^Ga-DOTATOC uptake by stellate ganglia. Only one similar case has been reported by Berg et al. using ^68^Ga-DOTATATE PET [19]. Another novelty of this case is the follow-up 3 months after the initial ^68^Ga-DOTATOC PET, which allowed to see a spontaneous decrease in the uptake. A recent review article also documented ^68^Ga-DOTATATE uptake, as the authors mentioned: “Low grade activity has also been described in cervical stellate ganglions. This is typically seen at the cervicothoracic junction para-vertebral and should not be interpreted as pathological lymph nodes” [20].

Comparatively, the uptake of prostate-specific membrane antigen (PSMA) ligands, such as ^68^Ga-PSMA-11 and ^18^F-PSMA-1007, by the stellate ganglia is a well-known phenomenon in PSMA PET imaging. This uptake, particularly in the cervical, celiac, and sacral ganglia, can be mistaken for lymph node metastases, potentially leading to diagnostic challenges [21]. Research indicates that PSMA ligand uptake in ganglia varies, with ^18^F-PSMA-1007 generally showing higher uptake than ^68^Ga-PSMA-11 due to differences in their physical properties and biodistribution. This variation highlights the importance of accurately distinguishing between physiological ganglia uptake and possible metastatic lesions to prevent the misinterpretation and incorrect staging of prostate cancer [22].

In our case, the uptake of the right cervical focus on ^68^Ga-DOTATOC PET/CT 1 was far less than that of the pancreatic primitive tumor and the liver metastasis, but we had considered the possibility of heterogeneity of the NET with less well-differentiated lymph node involvement leading to a decrease in uptake by ^68^Ga-DOTATOC. On the other hand, CT using RECIST criteria remains the standard of care for monitoring disease progression in pancreatic NETs [23]. However, the absence of lymph node involvement elsewhere made it unlikely that this right cervical focus corresponded to lymph node metastasis. Ultimately, the patient was considered to have stable disease and was not in need of a change in therapy.

Another main differential diagnosis was an enlarged parathyroid gland, especially in this case in the context of multiple endocrine neoplasia type 1 (MEN 1). Normal calcium and PTH levels exclude primary hyperparathyroidism, the most common manifestation of MEN1.

Furthermore, there were a few specific items that required discussion. Indeed, in managing PNETs, particularly in cases with advanced metastatic disease, careful consideration must be given to the balance between therapeutic efficacy and patient safety. Liver metastases, which are common in advanced PNETs, often dictate the therapeutic approach due to their impact on prognosis and treatment response. Multimodal strategies, including systemic therapy and liver-directed treatments, are typically tailored based on the extent of disease, the patient’s overall health, and tumor biology. The role of surgical intervention in such cases remains nuanced, particularly when extensive liver involvement poses heightened risks. This underscores the importance of an individualized, stepwise treatment plan aimed at optimizing outcomes while minimizing potential complications.

The decision to avoid initial surgical intervention in this patient was based on her extensive liver metastases and the associated risks of surgery in such scenarios. Additionally, her performance status was carefully considered (PS = 1, with mild asthenia), and it was determined that medical management with somatostatin analogs would offer the most immediate benefit while minimizing risks. While liver-directed therapies, including chemoembolization, are valuable options, they were deferred in this case until the efficacy of somatostatin analogs could be assessed. This stepwise approach aligns with the current management guidelines for advanced PNETs.

Regarding the tumor marker analysis, chromogranin A levels were significantly elevated (3000 ng/mL), as stated. Additional tumor markers, including serotonin and gastrin, were within normal limits, suggesting a non-functional tumor. This tumor was classified as non-functional based on the absence of hormone-related symptoms and the normal biochemical results for markers of functional NENs.

## 4. Conclusions

The stellate ganglia can pose a diagnostic challenge in ^68^Ga-DOTATOC PET imaging due to their variable radiotracer uptake. This variability, which can occur both between patients and within the same patient over time, may result from physiological factors or changes in SSTR expression. Occasionally, the uptake in the stellate ganglia is intense enough to mimic pathological findings, such as metastatic lymph nodes, potentially leading to false-positive diagnoses.

To avoid misinterpretation, clinicians must recognize this phenomenon and carefully assess the anatomical location of the uptake. Correlating PET findings with CT imaging and considering the patient’s clinical context are key steps in distinguishing normal physiological uptake from true pathological lesions. When uncertainty remains, follow-up imaging or additional diagnostic tests may be necessary to confirm the diagnosis and to guide appropriate management.

## Data Availability

The original contributions presented in the study are included in the article, further inquiries can be directed to the corresponding author/s.
